# The DLC-1 tumor suppressor is involved in regulating immunomodulation of human mesenchymal stromal /stem cells through interacting with the Notch1 protein

**DOI:** 10.1186/s12885-020-07542-5

**Published:** 2020-11-04

**Authors:** Tao Na, Kehua Zhang, Bao-Zhu Yuan

**Affiliations:** The Cell Collection and Research Center, National Institutes for Food and Drug Control, No. 2 Tiantan Xili, Dongcheng District, Beijing, 100050 China

**Keywords:** Notch1 protein, Immunomodulation, E3 ubiqitin ligase, Deleted in liver cancer 1 (DLC-1), Human mesenchymal stromal /stem cells (hMSCs), Hairy/enhancer-of-split related with YRPW motif protein 1 (Hey1)

## Abstract

**Background:**

Immunomodulatory activities of human mesenchymal stromal /stem cells (hMSCs) has been widely recognized as the most critical function of hMSCs for exerting its therapeutic effects. However, the detailed mechanisms responsible for regulating the immunomodulation of hMSCs still remain largely unknown. Previous studies revealed that the Notch1 protein exerted a pro-immunomodulatory function probably through interacting with the protein(s) subjective to proteasome-mediated protein degradation. The DLC-1 protein represents a well characterized tumor suppressor subjective to proteasome-mediated degradation. However, the detailed signaling pathway of Notch1 and the involvement of DLC-1 in regulating the immunomodulation of hMSCs have not been studied before.

**Methods:**

The transfection with cDNA or siRNA into hMSCs assisted by co-culture of hMSCs with peripheral blood mononuclear cells and small molecule inhibitors of signaling proteins, followed by immunoprecipitation, Western blotting, RT-PCR, and flowcytometry, were employed to characterize the Notch1 signaling, to identify DLC-1 as a candidate proteasome-targeted protein, and to characterize DLC-1 signaling pathway and its interaction with the Notch1 signaling, in the regulation of immunomodulation of hMSCs, specifically, the inhibition of pro-inflammatory CD4^+^-Th1 lymphocytes, and the release of immunomodulatory molecule IDO1.

**Statistical analysis:**

One-way ANOVA was utilized as a statistical tool to analyze the data presented as means ± SEM of at least three separate experiments.

**Results:**

The present study revealed that the Notch1-Hey1 axis, but not the Notch1-Hes1 axis, was likely responsible for mediating the pro-immunomodulatory function of the Notch1 signaling. The DLC-1 protein was found subjective to proteasome-mediated protein degradation mediated by the DDB1 and FBXW5 E3 ligases and served as an inhibitor of the immunomodulation of hMSCs through inhibiting Rock1, but not Rock2, downstream the DLC-1 signaling. The Notch1 signaling in the Notch1-Hey1 pathway and the DLC-1 signaling in the DLC-1-Rock1-FBXW5 pathway exhibited a mutual exclusion interaction in the regulation of immunomodulation of hMSCs.

**Conclusions:**

The present study uncovers a novel function of DLC-1 tumor suppressor in regulating the immunomodulation of hMSCs. It also proposes a novel mutual exclusion mechanism between the DLC-1 signaling and the Notch1 signaling that is possibly responsible for fine-tuning the immunomodulation of hMSCs with different clinical implications in hMSCs therapy.

**Supplementary Information:**

**Supplementary information** accompanies this paper at 10.1186/s12885-020-07542-5.

## Background

Human mesenchymal stromal /stem cells (hMSCs) is a group of fibroblast-like multipotent cells existing in almost all tissues with a limited self-renewal and differentiation potential to multiple cell lineages of endoderm, mesoderm and ectoderm [1]. The hMSCs of various tissue origins also exhibit unique immunomodulatory activities, which make hMSCs the most popular cell type used in stem cell-based therapies [2, 3]. To achieve the best clinical efficacy from hMSCs therapy, it is necessary to fully understand its immunomodulation, which represents the most important quality attribute of biological effectiveness of hMSCs.

The immunomodulation of hMSCs are manifested in part by their abilities to modulate almost all immune cells, such as T and B lymphocytes, natural killer cells [4], macrophages [5], and neutrophils [6]. During modulating CD4^+^ T lymphocytes, hMSCs inhibit proliferation and activity of pro-inflammatory lymphocytes, such as Type 1 T helper (Th1) and Type 17 T helper (Th17) subpopulations and promote polarization of regulatory T lymphocytes (Tregs) through cell-cell interaction and/or secretion of immunomodulatory molecules [7]. Among the key molecules, the Indoleamine 2,3-dioxygenase 1 (IDO1) protein still represents a major research interest of the immunomodulation of hMSCs [8].

IDO1 is a rate-limiting enzyme for catalizing tryptophan into kynurenine [9]. Its expression is induced by pro-inflammatory molecules, such as IFN-γ, TNF-α or IL-1α [10]. The importance of IDO1 in the immunomodulation of hMSCs has been established partially through employing either IDO1 silencing or small molecule IDO1 inhibitor, i.e. 1-methyl-L-tryptophan (1-L-MT). The IDO1 inhibition can cause significant reduction of various immunomodulatory activities of hMSCs, such as the reduction of inhibiting Th1 lymphocyte proliferation or promoting Treg polarization [8, 11]. However, even though the IDO1 activities have been characterized in substantial details, the molecular mechanisms, particularly the cell signaling pathways involved in regulating IDO1 activity, still remain largely unknown.

Among various signaling pathways, the Notch1 signaling has been previously revealed for promoting the immunomodulation of hMSCs [8]. The Notch1 signaling is activated sequentially through binding of Notch1 proteins to their ligands on surface of adjacent cells and two successive proteolytic cleavages mediated by TNF-α converting enzyme (TACE) and γ-secretase/presenilin complex [8]. Different types of γ-secretase inhibitors have been used as experimental tools to unveil novel functions of Notch signaling [12]. Among the most commonly used inhibitors is a small peptide inhibitor Gamma-secretase inhibitor I (GSI-I), which shares both structural and functional similarities with Botezomib, a proteasome inhibitor used frequently in cancer research [13]. The cleavage by γ-secretase results in release of the Notch1 intracellular domain (NICD1) from plasma membrane and the NICD1 translocation into the nucleus, where it binds the CBF1/RBP-Jk; Su(H)/Suppressor of Hairless; Lag-1 (CSL) protein complex and turns the complex from transcriptional repressor into transcriptional activator with consequent activation of downstream effectors of the Notch1 signaling [14].

Among the downstream effectors of Notch1, hairy and enhancer of split-1 (Hes1) and Hairy/enhancer-of-split related with YRPW motif protein 1 (Hey1) have been more intensely studied [15]. These two effectors may represent different aspects of the Notch1 signaling. For example, over-expression of Hey1, but not Hes1, induced over an 80-fold decrease in Collagen Type II Alpha 1 Chain (*Col2a1*) transcription in a 3-dimentional differentiation induction model, suggesting that the Notch signaling played an inhibitory role on chondrogenic differentiation, in which the Notch1-Hey-1 axis, rather than the Notch1-Hes1 axis, was most likely involved [16]. In addition, Hes1 and Hey1 might be differently involved in tissue development, whereas Hes1 was involved in the development of brain, skin and adipose tissues, Hey1 was associated with the development of heart and vasculature [17]. All these findings thus suggested that different Notch1 signaling axis may mediate different functional aspects of hMSCs.

The Deleted in Liver Cancer-1 (DLC-1) protein has been established as a tumor suppressor with abilities to inhibit growth, migration, invasion and metastasis of a large variety of common cancers [18–20]. While the DLC-1 gene expresses in almost all normal tissues, it is frequently absent or dramatically down-regulated in tumor tissues mainly due to genomic deletion and/or aberrant methylation at the promoter region of the gene [21]. In addition, the DLC-1 protein is subjected to cytoplasmic sequestration and proteasome-mediated degradation [22, 23], which may be governed by the CUL4A–DDB1–FBXW5 E3 ubiquitin ligase complex [24].

The DLC-1 protein is a multi-domain protein comprising of a Sterile Alpha Motif (SAM) domain, a Rho GTPase-Activating Protein (RhoGAP) domain and a StAR-related lipid-transfer (START) domain in its N-terminus, middle and C-terminus, respectively. In addition, it possesses a bipartite nuclear localizing sequence (NLS) responsible for DLC-1 protein nuclear translocation and a serine-rich region likely for regulating the NLS activity [23]. Among the major functional domains, the RhoGAP domain is highly conserved responsible for catalyzing hydrolysis of Guanosine-5′-triphosphate (GTP) into guanosine diphosphate (GDP) and subsequent inactivation of small Rho subfamily proteins, such as the Ras homologous A/B/C (RhoA/B/C) and Cell division control protein 42 homolog (Cdc42) proteins. A prominent downstream effector of the small Rho proteins is Rho-associated protein kinase 1/2 (Rock1/2), which transmit various activities of the Rho proteins in different types of cells [25].

In this study, we discovered a mutual exclusion crosstalk between the DLC-1 signaling and the Notch1 signaling in human umbilical-cord-derived mesenchymal stromal /stem cells (hUC-MSCs) following the search of candidate proteins subjective to proteasome-mediated protein degradation. The crosstalk detailed that the DLC-1 signaling in a way of FBXW5-DLC-1-Rock1 was inhibitory to the immunomodulation of hUC-MSCs and able to interact with the Notch1 signaling represented by the Notch1-Hey1 axis in a mutual exclusion manner, thus likely providing a fine-tuning mechanism in the regulation of immunomodulation of hMSCs.

## Methods

### Materials

Cells: hUC-MSCs was gifted anonymously from TuoHua Biotech company (Siping, China), where the cells were isolated and purified from Wharton’s Jelly of a discarded umbilical cord. The expression of the featured surface markers of hMSCs, the differentiation potentials to osteocytes, chondrocytes and adipocytes, and microbiological safety were tested for hUC-MSCs in our laboratory. Peripheral blood mononuclear cells (PBMCs) were freshly isolated using a conventional Ficoll method [26] from whole blood of healthy donors provided anonymously from local Red Cross. All data analysis associated with the use of hUC-MSCs and PBMCs in this study was conducted anonymously. **Antibodies:** the antibodies against IDO1, NICD1, DDB-1 and phosphor-STAT1 at Y701 (pSTAT1) were from Cell Signaling (Danvers, MA); the antibodies against DLC-1, from BD Bioscience (Franklin Lakes, NJ); the antibodies against ubiquitin, Hes1, Hey1, Rock1 and Rock2, from Santa Cruz Biotechnology (Dallas, TX); the antibodies against Cullin 4A and FBXW5, from Abcam (Cambridge, MA); the antibody against β-actin, from Sigma (Milwaukee, WI); Horseradish peroxidase (HRP)-conjugated anti-mouse or anti-rabbit secondary antibodies, from GE Healthcare (Piscataway, NJ). All antibodies conjugated with different fluorescent dyes were from BD Bioscience. **Constructs:** pIDO1-Luc, pcDNA3.1-DLC-1, −DLC-1-Δ622, −DLC-1-662, −DLC-1-R718E containing a R718E point mutation in RhoGAP domain, and -DLC-1-RhoGAP, which is the RhoGAP domain only mutant, were constructed in previous studies [8, 23]. **Chemicals:** GSI-I (SCP0004) and DAPT (D5942), γ-secretase inhibitors, were purchased from Sigma Aldrich (St. Louis, MI); Bortezomib, a proteasome inhibitor, was from ChemieTek (Indianapolis, IN); Y27632, a pan-Rock inhibitor, from EMD Millipore (Darmstadt, Germany); Phorbol-12-myristate-13-acetate (PMA) was from R&D (Minneapolis, MN), Ionomycin was from Santa Cruz Biotechnology (Dallas, TX), Brefeldin A (BFA) was from Abcam (Cambridge, MA).

### Detection of hMSCs surface markers

The hUC-MSCs surface markers, i.e. CD105, CD90 and CD73, were detected by flow cytometry using BD Stemflow hMSC Analysis Kit following the procedures described previously [8, 27].

### Osteogenic differentiation

The osteogenic differentiation of hUC-MSCs was examined by detecting the expression of Response Gene to Complement 32 protein (RGC32), an effective biomarker of osteogenesis revealed and validated in previous studies, via a real-time Polymerase chain reaction (PCR) following previously described procedures for the induction of osteogenic differentiation described previously [8].

### Semi-quantitative PCR

Conventional semi-quantitative RT-PCR was employed to detect mRNA expression of Hes-1 and Hey-1 in hUC-MSCs following various treatments. The expression of Glyceraldehyde 3-phosphate dehydrogenase (GAPDH) was used as an internal control for the semi-quantitation. The sets of primer sequences were: AGCACAGACCCAAGTGTGCTG and GAAGGTGACACTGCGTTGGG, for HES-1; ACGAGAATGGAAACTTGAGTTCGGC and CCCAAACTCCGATAGTCCATAGCAAG, for HEY-1; ACCACAGTCCATGCCATCAC and TCCACCACCCTGTTGCTGT, for GAPDH.

Real-time PCR with SYBR Green quantification were set up using 1/20 of each complementary DNA (cDNA) preparation in Applied Biosystems® 7500 Real-Time PCR Systems (Thermo Fisher scientific, Waltham, MA). Quantitative analysis was conducted by normalizing the expression level of the testing gene to that of GAPDH. The primer sequences were: GAAGTTCTGGGTCCTTTCATC and GCATGGATCGTCTGTTCTAATA, for RGC32; GGGCTGTGGAGTTTGGTGTC and CTGCTTGGGTGGGTGGAG, for Runx2; GATGGATTCCAGTTCGAGTATG and AGTGACGCTGTAGGTGAA, for Collagen I; TGCCTTTCCTGTAACGTTGGA and CCACAATGTTCTCTTCCCAAG, for Osterix; GGTCACTGATTTTCCCACGGA and TGGATGTCAGGTCTGCGAAAC, for Osteopontin; CTGACCACATCGGCTTTC and CAGATTCCTCTTCTGGAGTTTAT, for BGLAP; AGGCTGGAGAGGCGGCTAAG and TGGAAGGTGACACTGCGTTGG, for HES1; GGATCACCTGAAAATGCTGCATAC and CCGAAATCCCAAACTCCGATAG, for HEY1; GCCGGACACCATGATCCTAAC and GAGCCTCAATGGCATCTCTGT, for DLC-1; GTGTGAACCATGAGAAGTATGA and TAGAGGCAGGGATGATGTT, for GAPDH.

### Inhibition of Th1 lymphocyte proliferation

The effect of hUC-MSCs on inhibiting proliferation of Th1 lymphocytes from the PIB (acronym for PMA, Ionomycin and BFA)-induced PBMCs was examinedfollowing the previously reported procedures [8]. Briefly, fresh PBMCs were co-cultured with hUC-MSCs in 5:1 ratio for 18 h in RPMI 1640 complete medium containing 10% FBS and then stimulated with PIB (25 ng/ml PMA, 1 μg/ml Ionomycin and 10 μg/ml BFA) for another 5 h. Then, the PBMCs of all testing groups were collected and stained with both PerCP-Cy5.5-conjugated CD3 and FITC-conjugated CD8 for 30 min at room temperature. The PMBCs were then fixed with 4% paraformaldehyde fix solution for 15 min at room temperature. After that, the PBMCs were incubated with permeabilization medium containing PE-conjugated IFN-γ in dark for 30 min at room temperature. Finally, the PBMCs were washed twice with PBS, and analyzed using BD FACS Calibur flow cytometer. Using flow cytometry, the CD3 positive lymphocytes were gated first, the CD8^−^IFN-γ^+^ subpopulation was then identified as Th1 lymphocytes, the CD3^+^CD8^−^IFN-γ^+^ cells.

### Western blotting

The procedures of conventional Western blotting were followed to monitor changes in expression of relevant proteins in hUC-MSCs following various treatments [8].

### Immunoprecipitation (IP) for detecting the expression of poly-ubiquitinated proteins

The Cell lysates extracted using RIPA buffer from 1.5 × 10^6^ cells treated with GSI-I or Bortezomib were incubated with 1 μg antibody of the targeted protein for IP at 4 °C overnight, then incubated with protein A/G agarose at 4 °C for 1 h. After washing three times at room temperature, the agarose-bound cell lysates were then analyzed by Western blotting using ubiquitin antibody.

### Transient cDNA or small interfering RNA (siRNA) transfection

The procedures reported previously for either cDNA or siRNA transfection were followed for either over-expressing, or silencing the expression of the genes to be tested. Each plasmid DNA transfection was conducted using lipofectamine 2000-mediated transfection. To achieve equal transfection efficiency from different plasmid DNAs and to avoid biased results among different transfections in each individual experiment, all plasmid DNAs were extracted by the same laboratory personnel in the same time using endotoxin-free extraction kit following the same extraction and quality testing protocols. All plasmid DNAs used in each transfection experiment were carefully calculated to ensure all of them being in equal molar concentration. In addition, the transfection efficiency of each individual experiment was evaluated by Western blotting using β-actin as internal control to ensure equal amount of cells among different transfection groups were utilized in each individual experiment. The siRNA transfection was employed to silence the expression of Notch1, DLC-1, Hes-1 or Hey-1 in hUC-MSCs and the silencing effect for each target was determined by Western blotting or RT-PCR [8]. The siRNA sequences were CACCAGUUUGAAUGGUCAAtt for Notch1; AGAACAGCACCUCUGGGAUtt for DLC-1, CGAGGUGACCCGCUUCCUGtt (1#) [28] and AGACGAAGAGCAAGAAUAAtt (2#) [29] for Hes1, and GUGCGGACGAGAAUGGAAAtt (1#) and GACCGGAUCAAUAACAGUUtt (2#) for Hey1 [30]. The siRNA for Rock1 (sc-29,473) and Rock2 (sc-29,474) were purchased from Santa Cruz Biotechnology (Dallas, TX). The randomly scrambled siRNA was used as negative control.

### Construction of Notch1 and NICD expression vector

The pcDNA3.1-Notch1 containing full-length Notch1 cDNA was kindly provided by Dr. Jon C. Aster [31]. The cDNA of NICD1 was amplified by RT-PCR from total RNA of hUC-MSCs and constructed into the pcDNA3.1 expression vector. The primer sequences used for amplifying NICD were: 5′-GCTCTAGAGTGCTGCTGTCCCGCAAGCG-3′ and 5′-CCCAAGCTTTTCAACTTCCCTTCTCCAACATCATTTC-3′, in which Xbal and HindIII restriction sites were added for subcloning.

### Luciferase assay

The luciferase assay was employed for detecting IDO1 promoter activity. The pIDO1-Luc vector used for the assay was constructed and characterized in a previous study [8]. The procedures reported previously were followed for detecting IDO1 promoter activity in response to IFN-γ in hUC-MSCs [8].

### Data analysis

The data collected from the assays of cell viability, surface markers, luciferase-based promoter activity and Th1 lymphocyte proliferation were expressed as means ± SEM of at least three separate experiments. Comparison between group means was assessed using one-way analysis of variance with Newman–Keuls posttest (GraphPad Prism 4.0 Software, Inc., San Diego, CA, USA). The difference with *P* < 0.05 was considered significant.

## Results

### The treatment with GSI-I elevated DLC-1 protein level in hUC-MSCs through proteasome inhibition

**Fig. 1:**
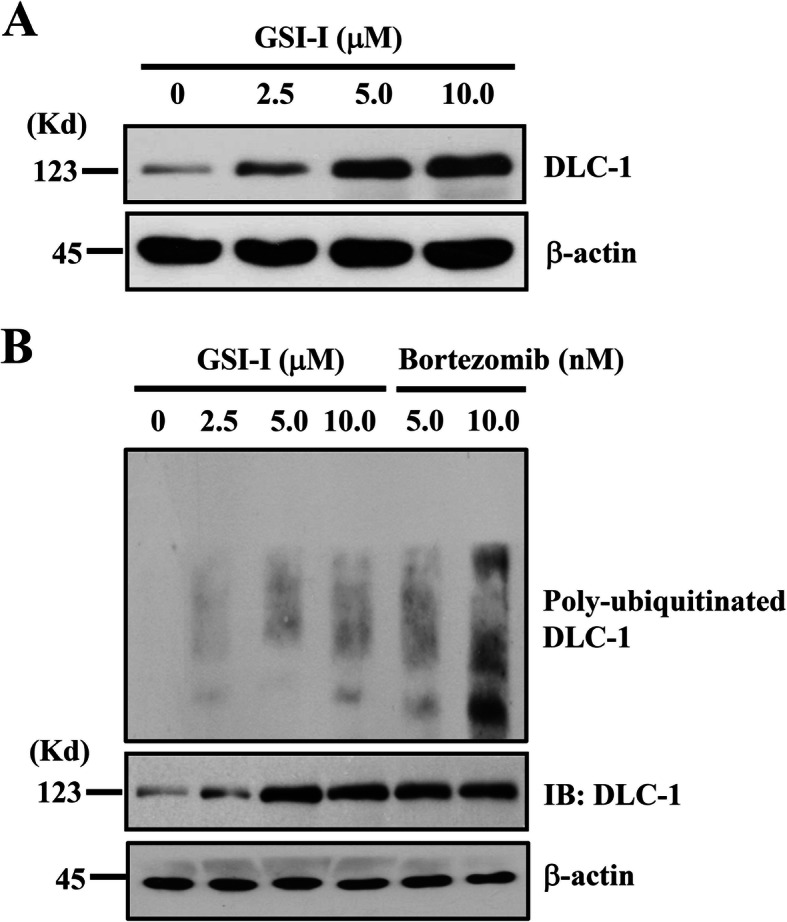
The treatment with GSI-I elevates DLC-1 protein level in hUC-MSCs through proteasome inhibition. Western blotting showed a dose-dependent increase in protein expression for DLC-1 at 24 h after GSI-I treatment (**a**), while the immunoprecipitation from the cell lysates using the antibodies against DLC-1 showed an increase in the polyubiquinated form of DLC-1 protein (**b**)

Given the dual inhibitory activities of GSI-I, we speculated from the previous study that the effect of GSI-I on the immunomodulation of hUC-MSCs was likely the consequence of interaction between Notch1 and other protein(s) that were subjected to proteasome-mediated protein degradation [8]. To identify the candidate proteins interacting with Notch1, we examined expression of different proteins likely undergoing proteasome-mediated degradation in hUC-MSCs. Among the candidate proteins were Mcl-1, DLC-1 and other proteins, which were subjective to the proteasome-mediated protein degradation [24, 32]. Through Western blotting and immunoprecipitation, we found that the GSI-I treatment significantly elevated DLC-1 protein level with an increase also seen in polyubiquitinated form of protein (Fig. 1a & b), suggesting that DLC-1 was subjected to the proteasome-mediated degradation in hUC-MSCs.

### The DLC-1 protein was involved in regulating the expression of surface markers of hUC-MSCs

**Fig. 2:**
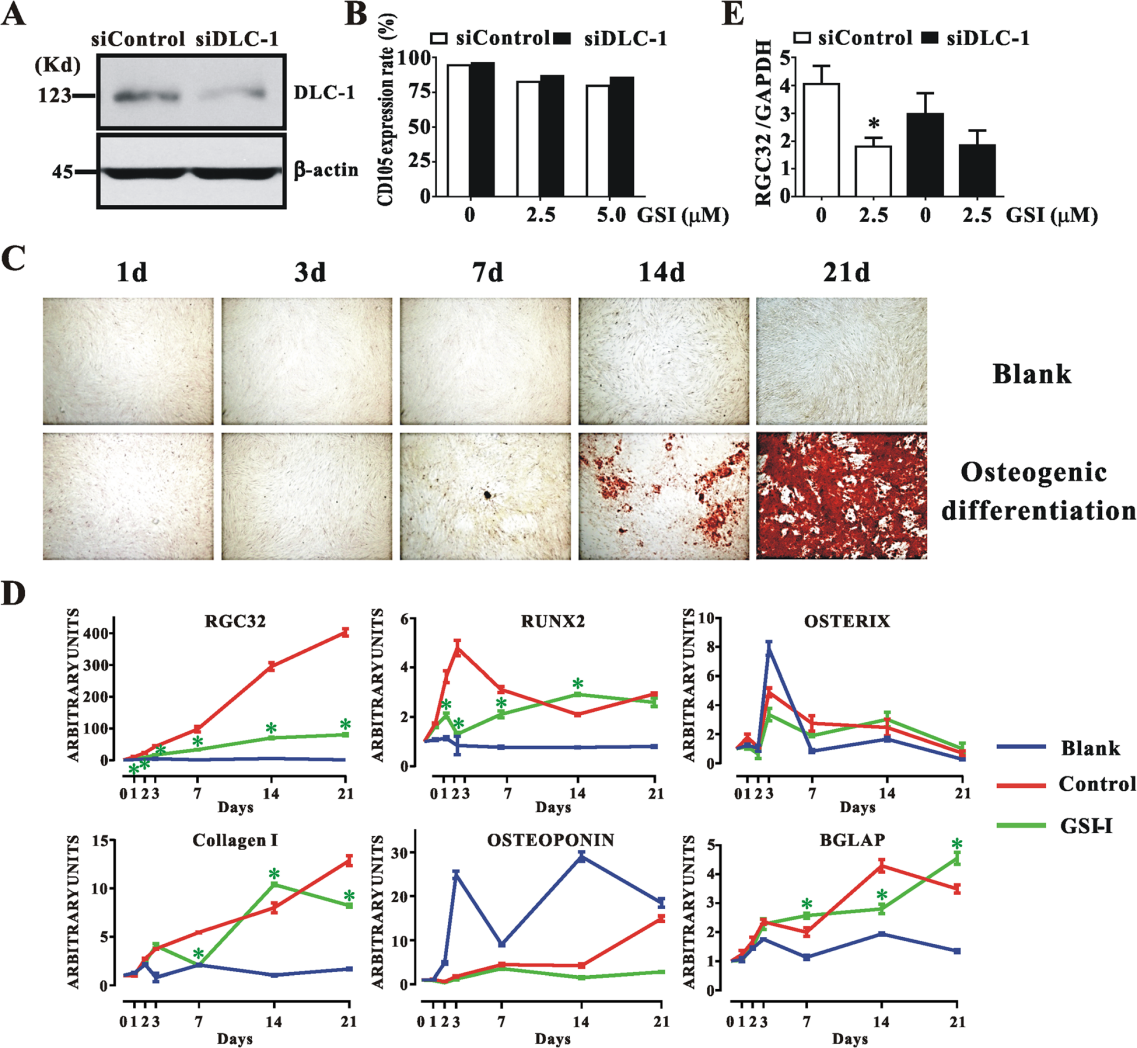
The DLC-1 protein is involved in regulating the expression of surface markers of hUC-MSCs. The siDLC-1 transfection effectively reduced DLC-1 protein expression as tested via Western blotting (**a**), moderately reversed the GSI-1-induced reduction of CD105 as tested via flow cytometry (**b**), but showed no effect on the expression of RGC32 gene as examined by RT-PCR (**c**)

Given that the Notch1 protein is involved in regulating the expression of the surface markers of hUC-MSCs, like CD73, CD90 and CD105, and osteogenic differentiation, which was measured in part by the transcription of RGC32, a surrogate marker of the osteogenesis of hUC-MSCs, revealed and validated in a previous study [8], to determine a possible involvement of DLC-1 in regulating surface markers and osteogenic differentiation of hUC-MSCs, we transfected hUC-MSCs with siDLC-1 in the presence of 2.5–5 μM GSI-I, then tested the expression of both the surface markers and RGC32. After confirming that the silencing effect from siDLC-1 transfection (Fig. 2a), we observed that, whereas the GSI-I treatment reduced the expression of CD73, CD90 and CD105 with the most significant reduction seen in CD105, the siDLC-1 transfection moderately reversed the GSI-I-induced reduction of all three surface markers (Fig. 2b). After compare the Alizarin red staining and osteogenic related gene marker determination using Realtime-PCR methods, we confirmed that RGC32 gene expression exhibited accumulatively increased along the entire osteogenic differentiation process. Therefore RGC32 is suitable for rapid determination during the early osteogenic differentiation step (Fig. 2c & d). The siDLC-1 transfection showed no effect on the inhibitory activity of GSI-I by RGC32 expression determination (Fig. 2e). Thus suggesting that the GSI-I-induced DLC-1 elevation contributed to the GSI-I-induced reduction in surface markers, but not in osteogenic differentiation.

### DLC-1 inhibited the immunomodulation of hUC-MSCs

**Fig. 3:**
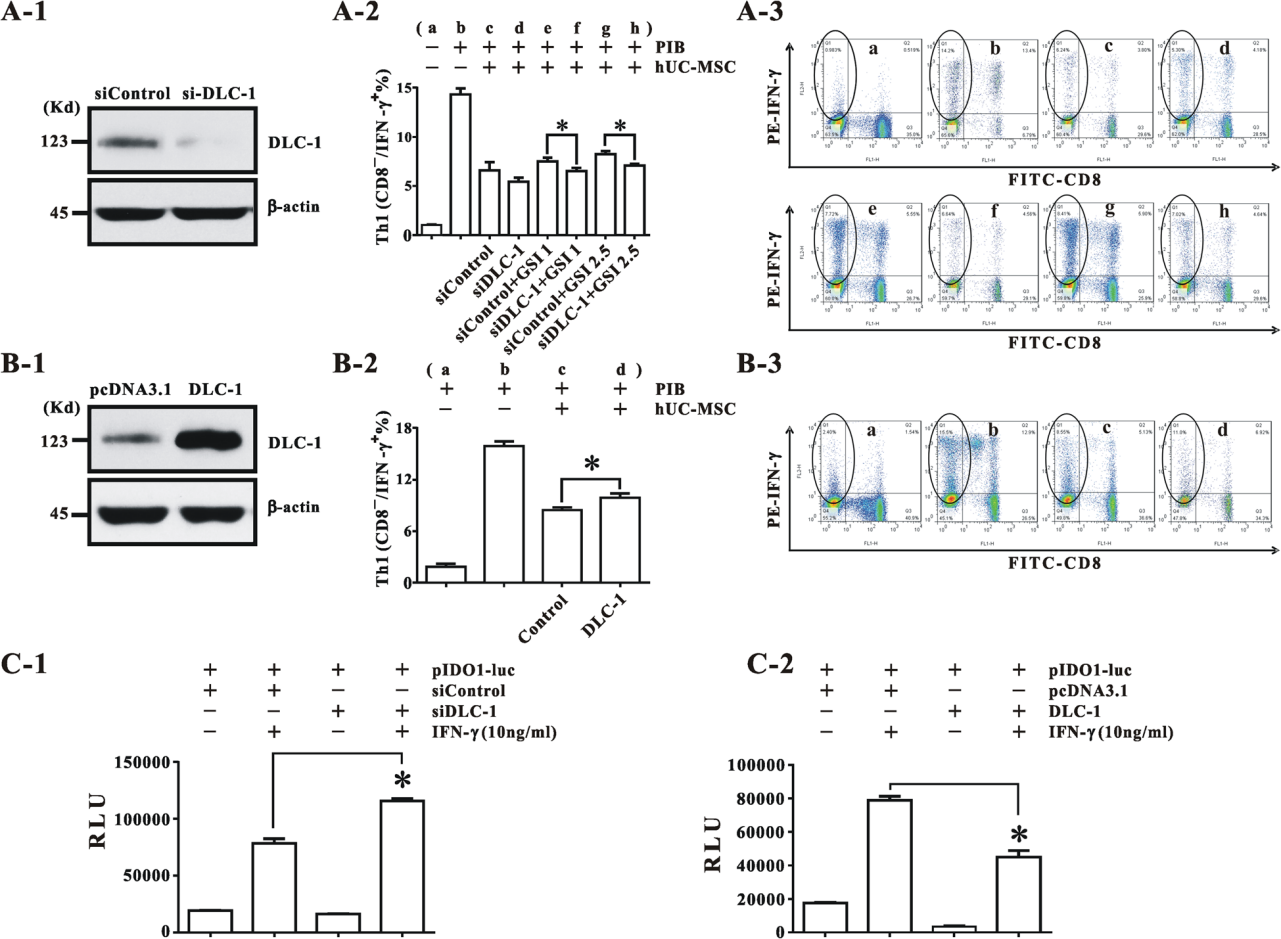
DLC-1 inhibits the immunomodulation of hUC-MSCs. **A** The DLC-1-silencing in hUC-MSCs, confirmed by Western blotting after siDLC-1 transfection (**A-1**), enhanced the inhibitory effect of hUC-MSCs with or without GSI-1 on Th1 lymphocyte proliferation, as tested via flow cytometry in Th1 lymphocyte proliferation assay (**A-2**). The original spectrogram of a representative Th1 lymphocyte analysis is presented (**A-3**). The Th1 lymphocytes are circled as the CD8^−^/IFN-γ^+^ cells in the spectrogram. All spectrograms are arranged in alphabetical order corresponding to the groups in **A-2**. **B** The DLC-1 overexpression from DLC-1 cDNA transfection in hUC-MSCs, confirmed by Western blotting (**B-1**), blunted the inhibitory effect of hUC-MSCs on Th1 proliferation, as tested via flow cytometry in the Th1 proliferation assay (**B-2**). PIB stands for PMA, Ionomycin and Brefeidin. The representative spectrogram of Th1 testing is presented (**B-3**). The CD8^−^/IFN-γ^+^ cells as circled in the spectrogram from the CD3-gated lymphocytes were recognized as Th1 lymphocytes. All spectrograms are arranged in alphabetical order corresponding to the groups in **B-2**. **C** The co-transfection of siDLC-1 with pIDO1-Luc in hUC-MSCs showed that DLC-1 silencing enhanced the IFN-γ-induced IDO-1 promoter activity. **D** The co-transfection of DLC-1 cDNA with pIDO1-Luc in hUC-MSCs indicated that DLC-1 over-expression inhibited the IDO1 promoter activity

Given that the immunomodulation of hMSCs can be represented in part by its inhibition of proliferation of Th1 lymphocyte subpopulation and IFN-γ-induced IDO1 expression [8], we transfected siDLC-1 or DLC-1 cDNA into hUC-MSCs and then examined the effect of the transfection on both Th1 proliferation and IDO1 expression. After confirming the effect of each transfection on DLC-1 expression (Fig. 3a & b), it was observed via flow cytometry assay that, whereas the siDLC-1 transfection further enhanced the reduction of Th1 proliferation and significantly reversed the GSI-I-induced inhibition of Th1 lymphocyte proliferation as well, the DLC-1 cDNA transfection significantly reduced the inhibition of Th1 proliferation (Fig. 3a & b). Meanwhile, through the IDO1 promoter assay, in which hUC-MSCs were co-transfected with pIDO1-Luc and siDLC-1, or pIDO1-Luc and DLC-1 cDNA for 24 h, as followed by the treatment with 10 ng/ml IFN-γ for another 24 h before measuring the luciferase activity from each transfection. It was observed that, comparing with each negative control, DLC-1 overexpression significantly reduced the IFN-γ-induced IDO1 promoter activity, whereas the DLC-1 silencing increased the promoter activity (Fig. 3c & d), all thus suggesting for the first time that DLC-1 played an inhibitory role in the immunomodulation of hUC-MSCs.

### The activity of DLC-1 inhibiting the immunomodulation of hUC-MSCs appeared to be both RhoGAP domain-dependent and RhoGAP domain-independent

**Fig. 4:**
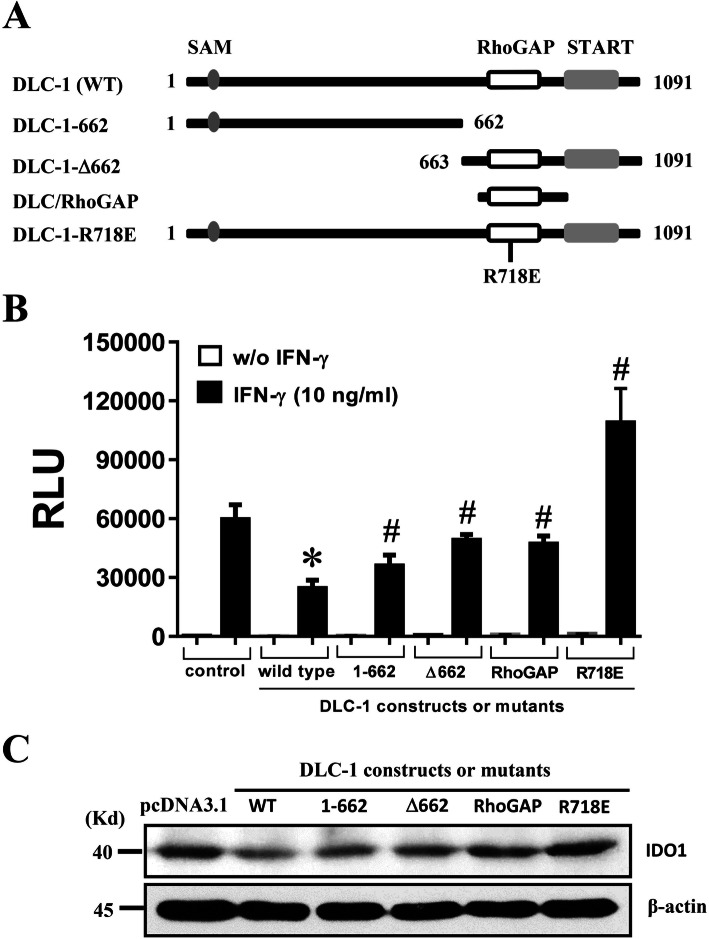
The DLC-1’s inhibitory effect on the immunomodulation of hUC-MSCs is both RhoGAP domain-dependent and RhoGAP domain-independent. **a** The schemes of all DLC-1 mutants used in this study are presented. **b** In the co-transfection of pIDO-1-Luc with the cDNA of wild type DLC-1 (wtDLC-1) or its mutants in hUC-MSCs, the wtDLC-1 showed an approximately 50 % reduction of the IFN-γ-induced IDO1 promoter activity, while all mutants exhibited either no effect (DLC-1-662) or even enhanced effect (DLC-1-662, −Δ662, −R714E and DLC-1/RhoGAP), on the promoter activity. **c** Western bloting showed in the transfection studies that the cDNA of wtDLC-1, DLC-1-662, or DLC-Δ662 caused a significant reduction in IDO1 expression, while the cDNA of DLC-1/RhoGAP showed no effect on, and the cDNA of DLC-1/RhoGAP resulted in, a slight increase in the IDO1 expression

To determine which functional domain(s) were responsible for DLC-1’s activity of inhibiting the immunomodulation, we then tested the effect of different DLC-1 mutants, i.e. the mutant with the deletion of N-terminus (DLC-1-Δ662) or C-terminus (DLC-1-622), the mutant with RhoGAP domain point mutation (DLC-1-R718E), or a RhoGAP domain only mutant, in comparison with wild type DLC-1, on the IFN-γ-induced IDO1 promoter activity in hUC-MSCs. The schemes of all DLC-1 mutants were shown in Fig. 4a. It was observed in the co-transfection of DLC-1 or its mutant cDNAs with pIDO1-Luc that wild type DLC-1 (wtDLC-1) inhibited over 50% of, the DLC-1-622 mutant showed no effect, and all other mutant showed even an increase in, the IDO1 promoter activity (Fig. 4b). In addition, the wtDLC-1 and the DLC-1-622 and DLC-1-Δ662 mutants reduced, but the DLC-1-R718E mutant increased, the IDO1 protein expression (Fig. 4c), all thus suggesting that the inhibitory effect of DLC-1 on IDO1 might be both RhoGAP domain-dependent and RhoGAP domain-independent.

### The effect of DLC-1 in hUC-MSCs was achieved through inhibiting the Notch1 signaling

**Fig. 5:**
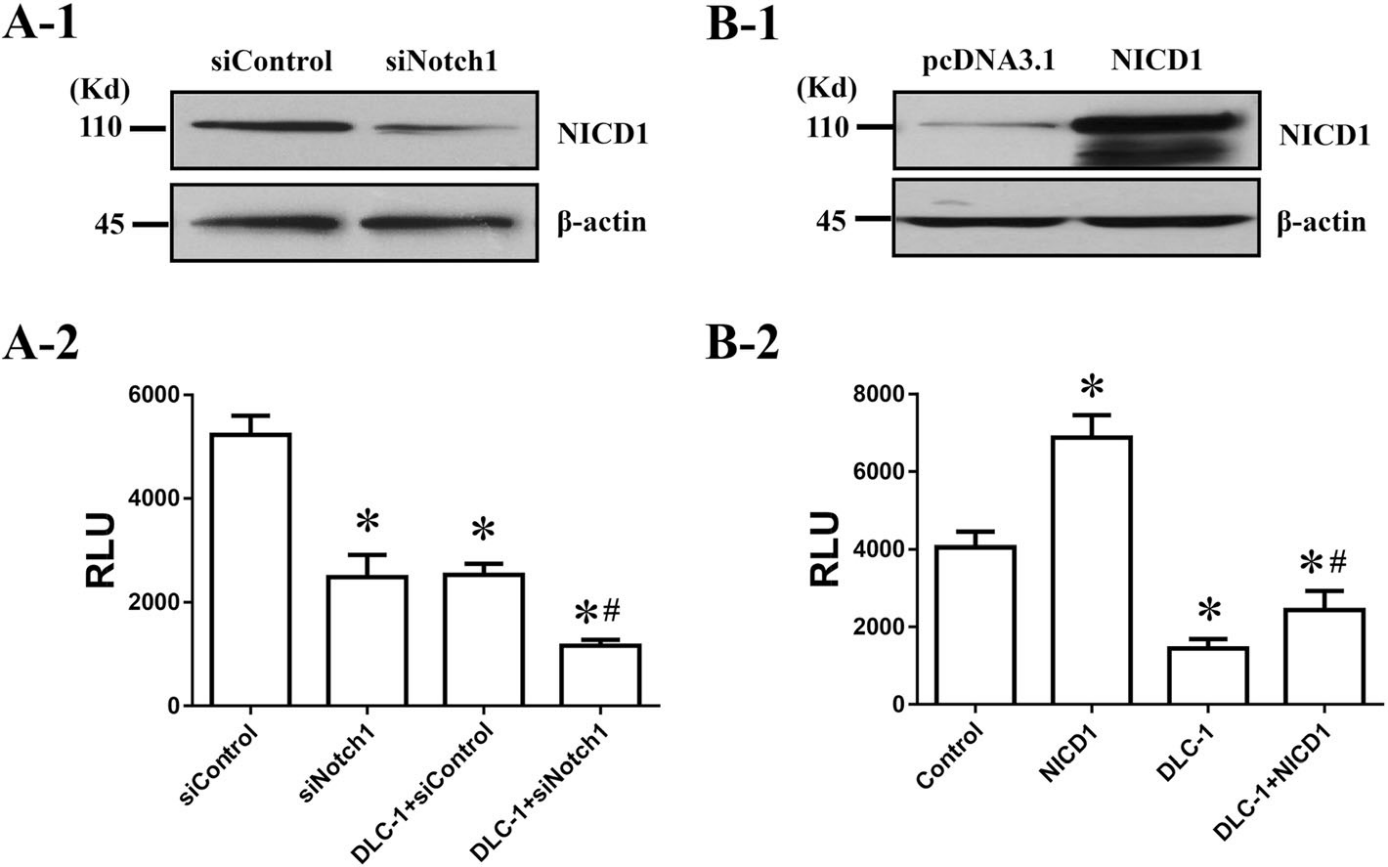
The effect of DLC-1 in hUC-MSCs is achieved through inhibiting the Notch signaling. **A** In the IDO1 promoter assay from the co-trasnsfection of DLC-1 cDNA with siNotch1, the Notch1 silencing was confirmed by Western blotting (**A-1**). While DLC-1 alone or siNotch1 alone reduced, the co-transfecton further inhibited, the IDO1 promoter activity (**A-2**). **B** In the IDO1 promoter assay from the co-trasnsfection of DLC-1 cDNA with NICD1 cDNA, the NICD1 over-expression was confirmed by Western blotting (**B-1**). DLC-1 inhibited the NICD1-elevated IDO-1 promoter activity (**B-2**). The IDO1 promoter activity was measured from the transfection with pIDO1-Luc followed by the treatment with IFN-γ

After associating the DLC-1 protein with the immunomodulation, we were then engaged to determine the association between DLC-1 and Notch1 protein regarding the regulation of immunomodulation of hUC-MSCs. To conduct this study, we first constructed the NICD1 expression vector according to the literature [31] and validated NICD1 expression in hUC-MSCs after transfecting the vector into the cells. Next, we tried to determine the association between DLC-1 and Notch1 in regulating IDO1 promoter activity after co-transfecting DLC-1 cDNA with either siNotch1 or NICD1 cDNA. It was found that, whereas the transfection with either siNotch1 or DLC-1 cDNA alone caused a similar reduction of the promoter activity, and siNotch1 plus DLC-1 cDNA further reduced the activity (Fig. 5a). Meanwhile, it was found that, whereas the NICD1 transfection alone caused a significant increase in the promoter activity, the effect was partially reversed by DLC-1 cDNA transfection (Fig. 5b), thus suggesting the existence of an association between DLC-1 and Notch1 at least in part in the regulation of IDO1.

### A mutual exclusion relationship existed between DLC-1 and Notch1 in hUC-MSCs

**Fig. 6:**
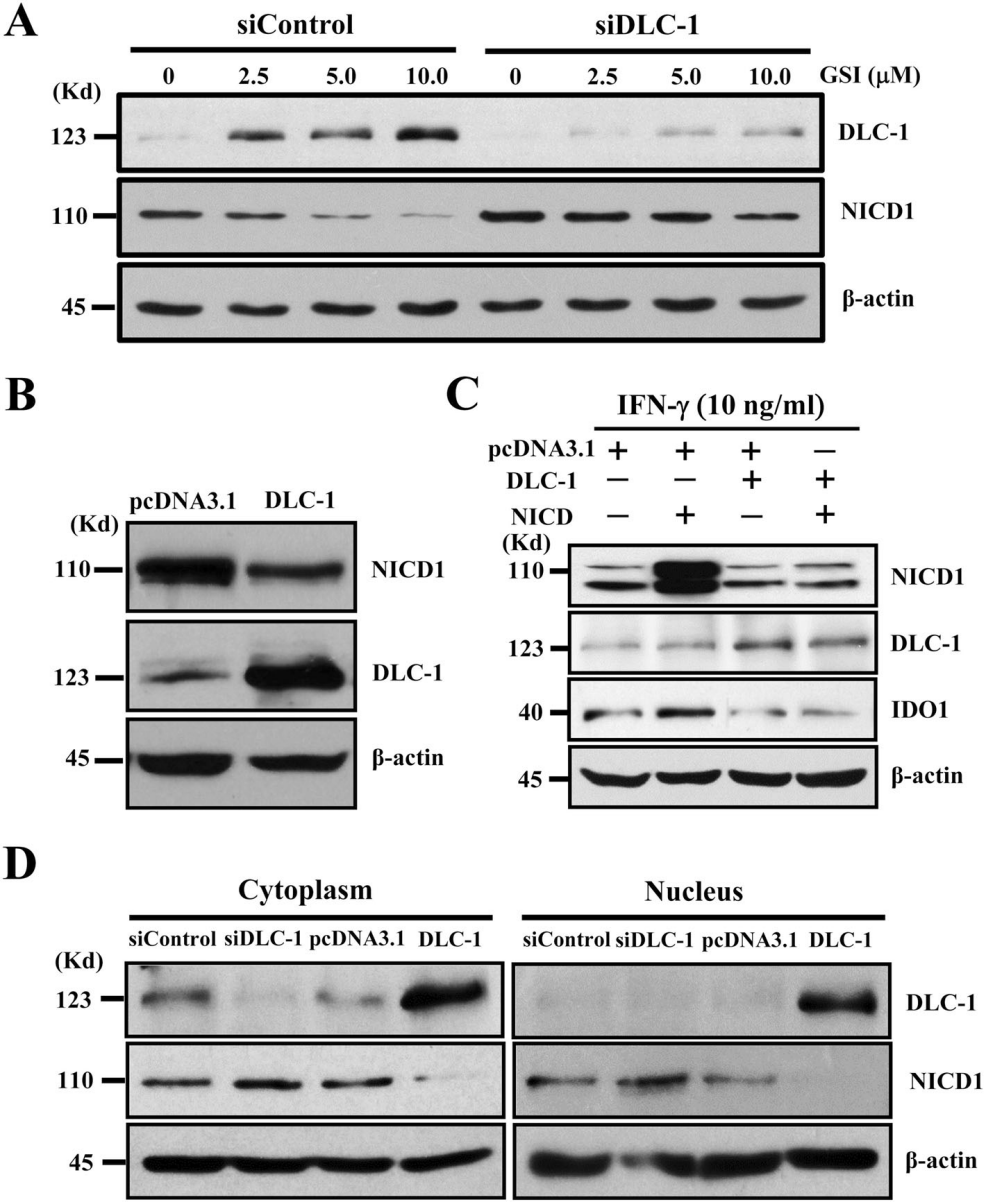
A mutual exclusion relationship exists between DLC-1 and Notch1 in hUC-MSCs. Western blotting showed that the siDLC-1 transfection reduced both basal and GSI-I-induced NICD1 (**a**); the transfection with DLC-1 cDNA caused a significant reduction of basal NICD1 level (**b**); in the co-transfection studies, whereas the transfection with NICD1 cDNA alone showed an over-expression of NICD1 and increases, and the transfection with DLC-1 cDNA alone reduced, the IDO1 expression, the co-transfection of both cDNAs inhibited NICD1 over-expression and the increased expression of IDO1 (**c**); in analyzing the partition in cellular compartments of DLC-1 and NICD1 proteins, the siDLC-1 transfection clearly increased the NICD1 level in both the cytoplasm and the nucleus, whereas the DLC-1 cDNA transfection reduced it in both cellular compartments with a more significant reduction seen in the nucleus than the cytoplasm (**d**). In all the experiments, the siControl and pcDNA3.1 were used as control in siRNA transfection and plasmid DNA transfection, respectively

To further reveal the relationship between DLC-1 and Notch1, we next examined the changes of Notch1 cleavage/activation in hUC-MSCs following the transfection with siDLC-1 [8]. Interestingly, it was found that the siDLC-1 transfection caused a significant increase in both basal NICD1 and the GSI-I-reduced NICD1 (Fig. 6a). Meanwhile, the transfection with DLC-1 cDNA alone resulted in a significant reduction of NICD1 (Fig. 6b). Furthermore, it was observed in the cDNA transfection experiments that, while the DLC-1 cDNA almost completely abolished the effect of inducing IDO1 by the NICD1 cDNA, the NICD1 cDNA blocked the effect of inhibiting IDO1 expression by the DLC-1 cDNA (Fig. 6c), all thus together strongly supporting the existence of a mutual exclusion relationship in protein expression between DLC-1 and Notch1 and on the regulation of IDO1.

Regarding the likely effect of DLC-1 on NICD1 nuclear translocation, we also revealed that, whereas the siDLC-1 transfection clearly increased -NICD1 protein level in both the cytoplasm and the nucleus, the DLC-1 cDNA transfection caused a significant reduction of NICD1 protein in both compartments with a more reduction seen in the nucleus than the cytoplasm (Fig. 6d), thus indeed suggesting that DLC-1 could also reduce NICD1 protein nuclear translocation.

### The Notch-Hey1 axis, but not the Notch-Hes1 axis, was involved in promoting the immunomodulation of hUC-MSCs

**Fig. 7:**
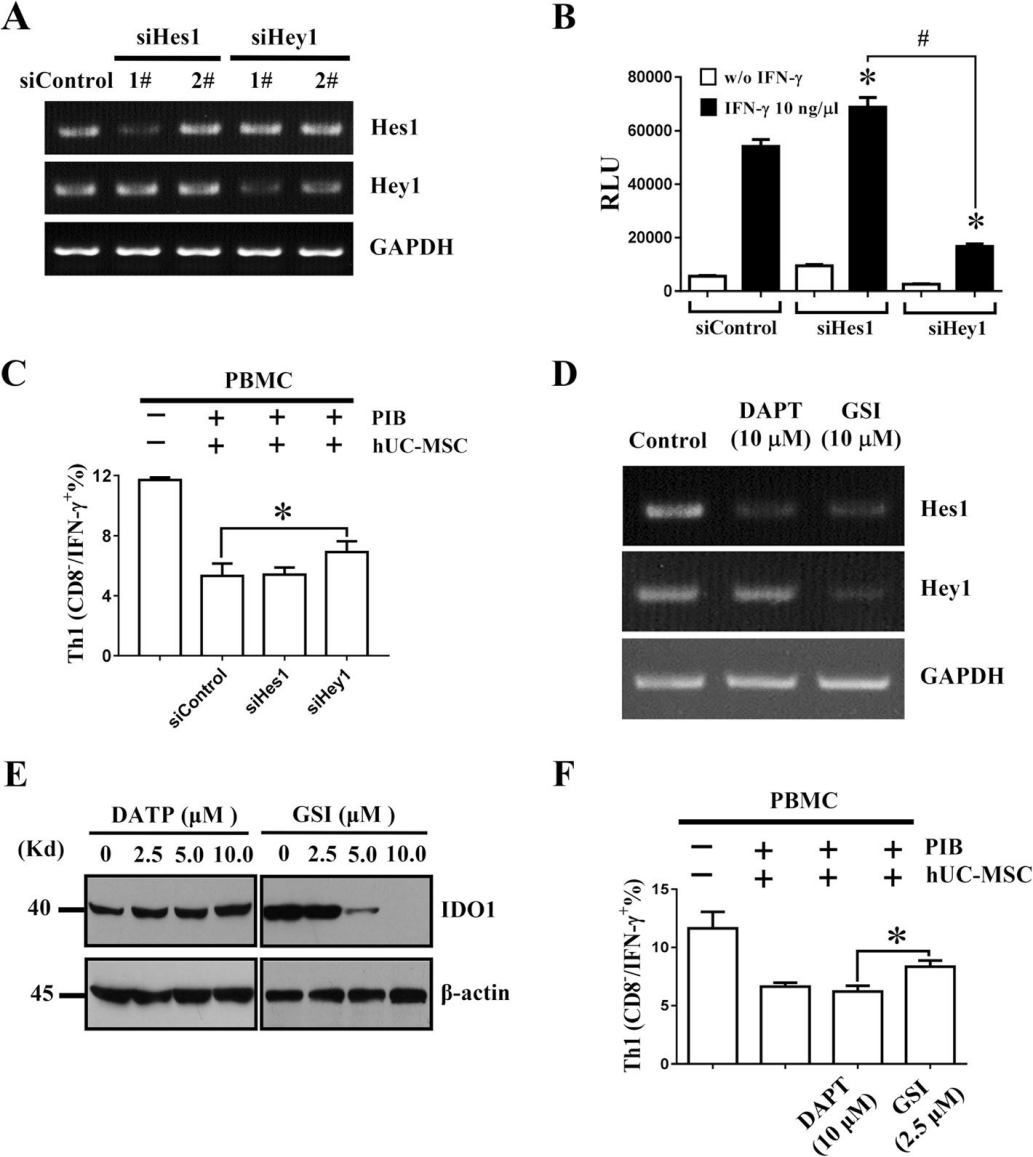
DLC-1 inhibits the activity of the Notch1-Hey1 axis, but not the Notch1-Hes1 axis, in the immunomodulation of hUC-MSCs. **a** RT-PCR validated that the transfection with siHes1 (1#) and siHey1 (1#) effectively inhibited the gene expression of Hes1 and Hey1, respectively. **b** The IDO1 promoter activity assay indicated that, comparing with siControl, the siHes1 transfection caused a slight increase, but the siHey1 transfection resulted in a dramatic decrease, in the IDO1 promoter activity. **c** The Th1 lymphocyte proliferation assay showed that the transfection with siHey1, but not siHes1, was able to significantly reduce the inhibition of Th1 proliferation by hUC-MSCs. **d** RT-PCR showed that the treatment with GSI-I equally reduced the gene expression of both Hes1 and Hey1, whereas DAPT only reduced that of Hes1. **e** Western blotting showed that DAPT caused a slight increase in, but GSI-I resulted in a significant decrease in, the IDO1 expression. **f** The Th1 lymphocyte proliferation assay indicated that GSI-I reduced, but DARP exhibited no effect on, the inhibition of Th1 lymphocyte proliferation by hUC-MSCs

Both Hey1 and Hes1 have been well characterized as two prominent downstream effectors of the Notch signaling, but may act differently in mediating different functional aspects of the Notch signaling [16, 33]. Therefore, it was of great interest to determine a possible distinction between these two effectors in mediating the Notch1-regulated immunomodulation of hUC-MSCs. By employing gene silencing with each specific siRNA followed by validation via RT-PCR, (Fig. 7a), we found that the Hes1 silencing caused a slight increase, but the Hey1 silencing resulted in a dramatic decrease, in the IFN-γ-induced IDO1 promoter activity (Fig. 7b). Meanwhile, it was also observed that it was the Hey1 silencing, but not Hes1 silencing, that was able to significantly reduce the inhibition of Th1 proliferation by hUC-MSCs (Fig. 7c), thus suggesting that it was the Notch1-Hey1 axis, but not the Notch1-Hes1 axis, that was involved in the immunomodulation of hMSCs.

Since it was reported that the Notch inhibitor DAPT exerted its inhibition more specifically on Hes1 than Hey1 [33], we then utilized DAPT as a tool in comparison with GSI-I to distinguish between Hes1 and Hey1 in the involvement in the immunomodulation of hMSCs. It was observed through RT-PCR that, while the treatment with 10 μM GSI-I equally reduced both Hes1 and Hey1 expression, the treatment with 10 μM DAPT only reduced Hes1 expression, thus confirming that DAPT was a Hes1-specific inhibitor (Fig. 7d). It was next observed that, whereas the GSI-I treatment reduced both IDO1 protein expression in hUC-MSCs and the inhibition of Th1 lymphocyte proliferation by hUC-MSCs, the DAPT treatment unexpectedly increased the IFN-γ-induced IDO1 protein expression and showed no effect on Th1 lymphocyte proliferation (Fig. 7e & f), thus supporting that it was the Notch1-Hey1 axis, but not Notch1-Hes1 axis, that promoted the immunomodulation of hUC-MSCs.

### The Hey1 protein served as a key molecule in mediating the mutual exclusion relationship between DLC-1 and Notch1

After charactering the differences between Hey1 and Hes1, we then attempted to determine whether the Hey1 protein was involved in the mutual exclusion relationship between DLC-1 and Notch1. We tested the expression of Hey1 and Hes1 genes using RT-PCR after transfection with either siDLC-1 or DLC-1 cDNA. It was found that the transfection with DLC-1 cDNA caused a slight reduction of Hes1, but a significant reduction of Hey1. In contrast, the siDLC-1 transfection resulted in a remarkable reduction of Hes1, but a significant elevation of Hey1 (Figure s1A, B & C). On the other hand, it was observed via Western blotting that, whereas the siHes1 transfection induced an apparent increase in IFN-γ-induced IDO1 expression but a clear decrease in DLC-1 expression, the siHey1 transfection induced a remarkable decrease in IDO1 expression, but a clear increase in DLC-1 expression (Figure s1D). Therefore, all these findings together clearly demonstrated that it was Hey1, but not Hes1, that served as the key signaling molecule involved in the mutual exclusion relationship between DLC-1 and Notch1.

### The Notch1-Hey1 axis regulates DLC-1 protein stability through modulating the expression of FBXW5 E3 ligase

Given that the DLC-1 protein could serve as a degradation target of the CUL4A-DDB1-FBXW5 E3 complex in tumor cells [24], it was of great interest to also determine the involvement of these E3-ligase proteins in regulating DLC-1 protein stability and in the relationship between DLC-1 and Notch1 in hUC-MSCs. For this purpose, we silenced the expression of each E3-ligase protein in hUC-MSCs, then determined the effect of each silencing on DLC-1 protein expression and on the immunomodulation of hUC-MSCs. Interestingly, after confirming the silencing effect of each E3 ligase by Western blotting (Figure s2A), we observed that the silencing of either DDB1 or FBXW5, but not CUL4A, significantly elevated DLC-1 protein level with a more significance seen in FBXW5 silencing. In addition, the silencing of either FBXW5 or DDB1 was accompanied by a significant reduction in IDO1 and p-STAT1 with a more significance seen again in FBXW5 silencing (Figure s2A). More interestingly, it was also observed that, comparing with DDB1 and CUL4A, the silencing of FBXW5 caused a significant reduction in the inhibition of Th1 lymphocyte proliferation (Figure s2B). These findings thus demonstrated that FBXW5 was the major E3 ligase for regulating DLC-1 protein stability and subsequent immunomodulation of hUC-MSCs.

To further pursue the possibility that the E3 ligase(s) for DLC-1 and Notch1-Hey1 signaling could be mutually regulated, we first silenced each E3 ligase and then tested its consequence on the expression of NICD1 and Hey1. We then found that, whereas the FBXW5 silencing led to a significant reduction in both NICD1 and Hey1, the silencing for DDB1 or CUL4A showed almost no such effect (Figure s2A). Next, we examined the effect of silencing Hes1 or Hey1 on the expression of all E3 ligases. It was then observed that, whereas the Hes1 silencing showed no effect on all E3 ligase proteins, the Hey1 silencing however caused a significant reduction only in FBXW5 protein, thus concluding that the Notch1-Hey1 signaling and FBXW5 could be mutually inhibitory for regulating DLC-1 protein stability and subsequent immunomodulation of hMSCs (Figure s2C).

### The inhibition of Rock1, but not Rock2, inhibited the immunomodulation of hUC-MSCs

Given that the Rock1/2 proteins serve as the key effectors of the DLC-1 signaling, we next attempted to determine whether they could play a role in regulating the immunomodulation of hUC-MSCs. In the new experiments, we employed Y27632, a Rock1/2 small molecule inhibitor, to treat hUC-MSCs and then tested the effect of the treatment on IFN-γ-induced IDO1 expression and IDO1 promoter activity in hUC-MSCs. Unexpectedly, we found that the treatment with Y27632 resulted in a significant dose-dependent increase in IDO1 protein expression and promoter activity (Figure s3A & B). To understand the seemingly contradictory effect of Y27632 on IDO1, we then tested via Western blotting the protein expression of Rock1 and Rock2, and the expression of DLC-1, NICD1, Hes1 and Hey1. Interestingly, it was found that the Y27632 treatment resulted in a dose-dependent reduction of both Rock1 and Rock2 with a much more significant reduction in Rock2. In addition, it also caused a dose-dependent decrease in DLC-1, but an increase in both NICD1 and Hey1 (Figure s3A). Moreover, consistent to the increase in IDO1, the hUC-MSCs pretreated with Y27632 exhibited a significantly enhanced inhibition of Th1 lymphocyte proliferation (Figure s3C), all thus suggesting that the treatment of Y27632 in fact mimicked the activity of the Notch1 signaling in enhancing the immunomodulation of hMSCs. Considering that Y27632 is an inhibitor of both Rock1 and Rock2, we next transfected either siRock1 or siRock2 before examining the expression of the relevant proteins, thus attempting to distinguish the effect between Rock1 and Rock2 in the immunomodulation of hMSCs. The results showed that the transfection with siRock2, but not siRock1, achieved the same effect as Y27632 on the expression of IDO1 and DLC-1, whereas the siRock1 transfection resulted in a clear reduction of IDO1 and unexpectedly an increase in Rock2 (Figure s3D), suggesting that the effect of Y27632 observed above on the immunomodulation of hMSCs was attributable to the inhibition of Rock2, but not Rock1, and Rock1 and Rock2 appeared exerting differently in the regulation of the immunomodulation with Rock1 seemingly being pro-immunomodulatory and Rock2 anti-immunomodulatory. It could be further suggested that Rock1 likely represented the inhibitory target downstream of the DLC-1 signaling in the inhibition of immunomodulation of hUC-MSCs, while Rock2 might serve as a negative feedback regulator of the DLC-1 signaling in this perspective.

## Discussion

The unique immunomodulatory activities provide hMSCs a great versatility in managing various inflammatory/immune situations for treating a large variety of uncontrolled inflammatory or abnormal immune diseases. It is then reasonable to believe that the fine-tuned regulatory mechanisms yet to be identified must be always available for ensuring hMSCs to effectively sense various inflammatory environments for precisely modulating the corresponding inflammations utilizing precisely oriented regulatory capacities. Therefore, the endeavor to understand the fine-tuned mechanisms endowing such versatility is extremely important for fully appreciating the therapeutic effects of hMSCs. With a set of novel evidence, the present study demonstrates that a crosstalk between two important cell signaling represents a means of fine-tuning the immunomodulation of hMSCs.

The two signaling revealed in this study are the Notch1-Hey1 signaling and the FBXW5-DLC-1-Rock1 signaling, which regulate the immunomodulation of hMSCs in opposite directions. Whereas the Notch1-Hey1 signaling promotes, the FBXW5-DLC-1-Rock1 signaling inhibits, the immunomodulation of hMSCs. More importantly, the activities of these two signaling are mutually exclusive, thus providing a means of fine-tuning the immunomodulation of hMSCs.

The mutual exclusion mechanism presented in this study is built up on a hypothesis proposed previously that some protein(s) subjective to proteasome-mediated protein degradation may antagonize the Notch1 signaling in regulating the immunomodulation of hUC-MSCs [8]. Although the search for DLC-1 represents the authors’ preferential long-term interest, the result from the search indeed supports that DLC-1 serves as a good candidate fitting the hypothesis.

The DLC-1 tumor suppressor was first revealed in this study as a potential modulator of the surface markers of hMSCs, the CD105 in particular, suggesting it may be associated with overall quality of hMSCs, as CD105 is involved in multiple functions of hMSCs, including osteogenic differentiation [8, 34], angiogenesis [35], and regenerative/therapeutic potential [36]. However, the most important finding about DLC-1 in this study was the interaction between DLC-1 signaling and Notch1 signaling in regulating the immunomodulation of hMSCs.

From thoroughly analyzing the key members of each signaling and their involvement in the relationship between DLC-1 and Notch1, a sophisticated mutual exclusion mechanism between the two signaling then emerges.

On the side of the DLC-1 signaling, this study reveals for the first time that the DLC-1 tumor suppressor is able to inhibit the immunomodulation of hUC-MSCs. This novel activity of DLC-1 is supported by the evidence that the change in DLC-1 expression is directly associated with the change of IFN-γ-induced IDO1 expression and the inhibition of Th1 lymphocyte proliferation by hUC-MSCs (Fig. 3). In this regard, the activity of DLC-1 appears to be both RhoGAP domain-dependent and RhoGAP domain-independent, although the structural integrity of the protein is needed (Fig. 4). Nevertheless, this novel finding may be of great importance as hMSCs exist abundantly in tumor microenvironment and contribute to the immunosuppression for tumor progression, especially the metastatic progression [37]. If DLC-1 can inhibit the immunosuppression of hMSCs, it then exerts its tumor suppressor functions on both seeds (tumor cells) and soil (mesenchymal cells) in tumor microenvironment according to the ‘seed-soil’ theory of tumorigenicity [38].

When functioning in hMSCs in the association with treating inflammatory diseases, the regulation of DLC-1 by proteasome-mediated protein degradation, rather than genomic deletion and/or epigenetic changes, may provide a flexible mechanism allowing DLC-1 to exert its activity in hMSCs on the basis of necessity. In comparison, genomic deletion and/or aberrant methylation occurring frequently for DLC-1 in different human cancers represent the more rigid mechanism(s) necessary for tumor cells to achieve a complete knock-down of the tumor suppressor functions of DLC-1.

Concerning the composition of the E3 ligase complex responsible for degrading DLC-1 protein, our study suggests that the complex is composed mainly of the DDB1 and FBXW5 E3 ligases in hMSCs. However, in a set of lung cancer cells, the complex responsible for degrading DLC-1 comprises CULT4, DDB1 and FBXW5 E3 ligases all together, thus suggesting the difference existing in the composition of E3 ligases between cancer cells and hMSCs [24]. This notion is supported by a study, in which DDB1 and FBXW7, which is in the same family with FBXW5, is sufficient to form an E3 ligase complex without CUL4A for degrading MYC proteins [39], supporting that two E3 ligases, i.e. DDB1 and FBXW5, rather than three ligases are sufficient for mediating DLC-1 degradation in hMSCs. The discrepancy between lung cancer cells and hUC-MSCs in the composition of E3 ligase proteins may represent different regulation stringencies needed in different types of cells. The regulation for DLC-1 protein stability may be more stringent in parenchymal cells with three E3 ligases than that in mesenchymal cells using two E3 ligases, thus providing more flexibility for DLC-1 in regulating the immunomodulation of hMSCs.

On the side of the Notch1 signaling, we first advanced our understanding about the involvement of Notch1 in immunomodulation of hMSCs by demonstrating that the Notch1 signaling diverges at the level of Hey1 and Hes1 regarding the immunomodulation of hMSCs. The new evidence revealed from the experiments employing either gene silencing or γ-secretase inhibitors GSI-I and DAPT suggest that the Notch1-Hey1 axis, rather than the Notch1-Hes1 axis, is likely involved in the immunomodulation of hMSCs (Figs. 7 and s1). As the Notch signaling possesses a large variety of functions, the distinguished roles between Hey1 and Hes1 downstream of the Notch1 signaling are of highly realistic relevance because different downstream effectors of any functionally diverse signaling need to represent different functional perspective of the signaling. More importantly, the distinction between Hey1 and Hes1 provides a sophisticated multi-level basis on the side of the Notch1 signaling for establishing the mutual exclusion relationship between Notch1 and DLC-1.

The multi-level mutual exclusion relationship between the two signaling is identified by employing a general approach, in which the function of the key molecule of each signaling was characterized for its association with the function of the opposite signaling in terms of regulating the immunomodulation of hMSCs. Through the characterization, each of the DLC-1, FBXW5 and Rock1 proteins of the DLC-1 signaling showed the ability to inhibit the expression of NICD1 and Hey1 and the associated immunomodulation of hMSCs (Fig. 7). Similarly, both Notch1 and Hey1 were demonstrated for their activity of reducing the expression of DLC-1, but inducing the expression of FBXW5 (Figure s2). Interestingly, although both FBXW5 and DDB1 are directly involved in regulating DLC-1 protein stability, it is FBXW5, but not DDB1, that is directly involved in the mutual exclusion relationship, thus leading to a possibility that, within the E3 ligase complex responsible for degrading DLC-1, it is the FBXW5 protein that is more likely to act as a sensor for detecting the degradation signal released from the Notch1 signaling.

The characterization also leads to a conclusion that the mutual exclusion relationship between the DLC-1 and Notch1 signaling is formed by the intra-signaling and inter-signaling transduction. The intra-signaling transduction along the DLC-1 signaling is in the order of FBXW5-DLC-1-Rock1. The inter-signaling transduction concerns mutual crosstalk between the two signaling; the crosstalk can be initiated at multiple levels of each signaling, transducted through the intra-signaling, released via the inter-signaling, and then sensed or received by the opposite signaling. It is thus imaginable that this type of crosstalk can provide a basis for achieving a mutual inhibition/exclusion of the two signaling regarding the regulation of the immunomodulation of hMSCs. For example, the inter-signaling transduction initiated in the Notch1 signaling can be transduced to the Hey1 protein and then released to the FBWX5 protein of the DLC-1 signaling for achieving the enhanced immunomodulation through inhibiting the DLC-1 signaling. With both inter- and intra- signaling transduction, the multi-level mutual exclusion mechanism can then provide a sophisticated automation system for fine-tunning the immunomodulation of hMSCs. The importance of this fine-tuned system is to set up a constantly dynamic control to sense and meet different needs of immunomodulation in various inflammatory environments.

With the framework about the crosstalk between the two signaling being proposed, a set of preliminary data provided in this study also suggests the existence of negative feedback control mechanism within each signaling for regulating the activity of that signaling. Within the DLC-1 signaling, the Rock2 protein may serve as a negative feedback molecule for limiting the activity of the DLC-1 signaling as the Rock2 inhibition induced by siRock2 or Y27632, which exhibits a more preferential inhibition on Rock2 than Rock1 in our experiments, inhibits DLC-1 protein expression while elevating IDO1 expression (Figure s3). Similarly, within the Notch1 signaling, the Hes1 protein may serve as a negative feedback molecule for inhibiting the Notch1 signaling as the Hes1 silencing elevates NICD1 and IDO1, but decreases DLC-1, an apparently opposite effect to the Hey1 silencing. The negative feedback control branched from the downstream effector of each signaling represents a common and effective control mechanism seen in different cell signaling pathways [40] and provides an additional level of the sophistication to the mutual exclusion crosstalk. Nevertheless, further investigation for the importance of the negative feedback mechanisms is warranted.

While more mechanistic studies are needed for fully understanding the mutual exclusion mechanism, one previous observation about the association of Caveolin-1 with the activity of γ-secretase may help interpret the signaling transduction from DLC-1 to Notch1. The DLC-1 protein can bind the Caveolin-1 protein in a START domain-dependent manner [41, 42]. The binding may lead to a negative regulation of γ-secretase activity as the Caveolin-1 protein is involved in the attenuation of γ-secretase-mediated proteolysis of Notch1 [43]. Nevertheless, further investigations are warranted to address all relevant details mediating the crosstalk between the two signaling before fully understanding the mutual exclusion crosstalk between them.

Besides the future mechanistic studies for further advancing our understanding of the mutual exclusion mechanism, the present study in fact inspires several interesting thinking which could be potentially exploited for future development of new testing methods to evaluate the quality of hMSCs or for the design of novel approaches to enhance therapeutic efficacy of hMSCs. For example, the key molecules involved in the mutual exclusion relationship may be developed as novel DLC-1/Notch1-based or FBXW5/Hey1-based surrogate markers for evaluating the versatility of immunomodulation of hMSCs; Rock2-specific inhibitors may be included in novel priming/licensing strategies to enhance therapeutic efficacy of hMSCs or may be utilized as small molecule therapeutics to provoke immunomodulatory potential of endogenous hMSCs in future ‘cell-free’ hMSCs therapy.

## Conclusions

In summary, the present study significantly advances our understanding about the regulation of the immunomodulation of hMSCs. It demonstrates for the first time that the DLC-1 tumor suppressor can function as an inhibitor of the immunomodulation of hMSCs, thus further emphasizing the importance of the DLC-1 tumor suppressor in regulating both tumor ‘seed’ and tumor ‘soil’, both of which are critical for tumor development and progression according to the ‘seed-soil’ theory of tumorigenicity [38]. On top of the findings about DLC-1, the present study in fact discovers a sophisticated multi-level mutual exclusion mechanism, which well fits the need of fine-tuning the immunomodulation of hMSCs. It is believed that the findings are of great potential for developing new quality evaluation biomarkers, newly designed hMSCs products or new therapeutic modalities.

## Supplementary Information

**Additional file 1: Figure S1.** Hey1, but not Hes1, mediate the mutual exclusive relationship between DLC-1 and Notch1. A-C: RT-PCR showed that the transfection with siHes1 or siHey1 can effectively reduce the gene expression of Hes1 (A-1) or Hey (A-2), respectively; the transfection with DLC-1 cDNA caused a slight reduction of Hes1 expression (B-1), but a significant reduction of Hey1 expression (B-2). In contrast, the siDLC-1 transfection resulted in a remarkable reduction of Hes1 expression (C-1), but a significant elevation of Hey1 expression (C-2). D. Western blotting showed that, while the siHes1 transfection induced an apparent increase in the expression of both IDO1 and NICD1, but a clear decrease in DLC-1 expression, the siHey1 transfection induced a remarkable decrease in the expression of both IDO1 and NICD1, but a clear increase in DLC-1 expression.

**Additional file 2: Figure S2.** The Notch1-Hey1 axis regulates DLC-1 protein stability through modulating the expression of CULT4-DDB1-FBXW5 E3 ubiquitin ligase complex. A. Western blotting confirmed that the transfection with siCULT4, siDDB1 or siFBXW5 can effectively silence the protein expression of each target gene. The silencing of both DDB1 and FBXW5, but not CUL4A, resulted in a significant increase in DLC-1 level and a significant decrease in IFN-γ-induced IDO1 and p-STAT1 level with the silencing of FBXW5 exhibiting more significant effect than that of DDB1. The FBXW5 silencing caused in a significant decrease in both NICD1 and Hey1, while the siDDB1 silencing showed only a slight reduction of NICD1 but an increase in Hey1, and the CUL4A silencing exhibits no effect on both NICD1 and Hey1. B. The Th1 lymphocyte proliferation assay showed that the FBXW5 silencing, but not the silencing of either DDB1 or CUL4A, resulted in a significant reduction of the inhibition of Th1 lymphocyte proliferation by hUC-MSCs. C. Western blotting showed that, while the Hes1 silencing exerted no effect on the expression of CUL4A, DDB1 and FBXW5, the si-Hey1 silencing resulted in a significant reduction of FBXW5 only.

**Additional file 3: Figure S3.** The Y27632-induced Rock2 inhibition, but not Rock1 inhibition, promotes the immunomodulation of hUC-MSCs probably through inhibiting the DLC-1 signaling while elevating the Notch1 signaling. A. Western blotting showed that the treatment with Y27632 for 24 h resulted in a dose-dependent reduction of both Rock1 and Rock2 with a much more significance seen for Rock2 as accompanied by the reduction of DLC-1 and Hes1. The treatment also resulted in a significant dose-dependent increase in the IFN-γ-induced IDO1 expression, as accompanied by the increase in both NICD1 and Hey1. B. The promoter activity assay showed that the Y27632 treatment caused a dose-dependent increase in IDO1 promoter activity. C. The Th1 lymphocyte proliferation assay showed that the pretreatment with Y27632 for 24 h exhibited a clear increase in the inhibition of Th1 lymphocyte proliferation with the statistical significance seen in the pretreatment with 10 μM Y27632. D. Western blotting showed that the silencing of Rock2, but not Rock1, exhibits the same effect as Y27632 on the expression of IDO1, DLC-1, NICD and Hey1.

## Data Availability

The datasets generated in and/or analyzed from the current study are available from the corresponding author upon reasonable request.

## Abbreviations

1-L-MT: 1-methyl-L-tryptophan
Brefeldin A: BFA
Cdc42: Cell division control protein 42 homolog
cDNA: Complementary DNA
Col2a1: Collagen Type II Alpha 1 Chain
CSL: CBF1/RBP-Jk; Su(H)/Suppressor of Hairless; Lag-1
CUL4A: Cullin-4A
DDB1: Damage Specific DNA Binding Protein 1
DLC-1: Deleted in liver cancer 1
FBXW5: F-box/WD repeat-containing protein 5
GAPDH: Glyceraldehyde 3-phosphate dehydrogenase
GDP: Guanosine diphosphate
GTP: Guanosine-5′-triphosphate
Hes1: Hairy and enhancer of split-1
Hey: Hairy/enhancer-of-split related with YRPW motif protein 1
hMSC: Human mesenchymal stem/stromal cells
HRP: Horseradish peroxidase
hUC-MSCs: Human umbilical-cord-derived mesenchymal stem/stromal cells
IDO1: Indoleamine 2,3-dioxygenase 1
IL-1a: Interleukin-1 alpha
IFN-γ: Interferon-gamma
IP: Immunoprecipitation
NICD: Notch intracellular domain
NLS: Nuclear localizing sequence
PBMCs: Peripheral blood mononuclear cells
PCR: Polymerase chain reaction
RGC32: Response gene to complement 32 protein
Rho: Ras homologous
RhoGAP: Rho GTPase-activating protein
Rock1/2: Rho-associated protein kinase 1/2
RT-PCR: Real-time Polymerase chain reaction
SAM: Sterile alpha motif
siRNA: Small interfering RNA
START: StAR-related lipid-transfer
STAT1: Signal transducer and activator of transcription 1
TACE: TNF-α converting enzyme
Th1: Type 1 T helper
Th17: Type 17 T helper
TNFα: Tumor necrosis factor alpha
Treg: Regulatory T lymphocytes
