# Supplementary figures and images for: The DLC-1 tumor suppressor is involved in regulating immunomodulation of human mesenchymal stromal /stem cells through interacting with the Notch1 protein
Source: BMC Cancer. 2020 Nov 4;20:1064. doi: 10.1186/s12885-020-07542-5 (PMC7640439; doi:10.1186/s12885-020-07542-5)

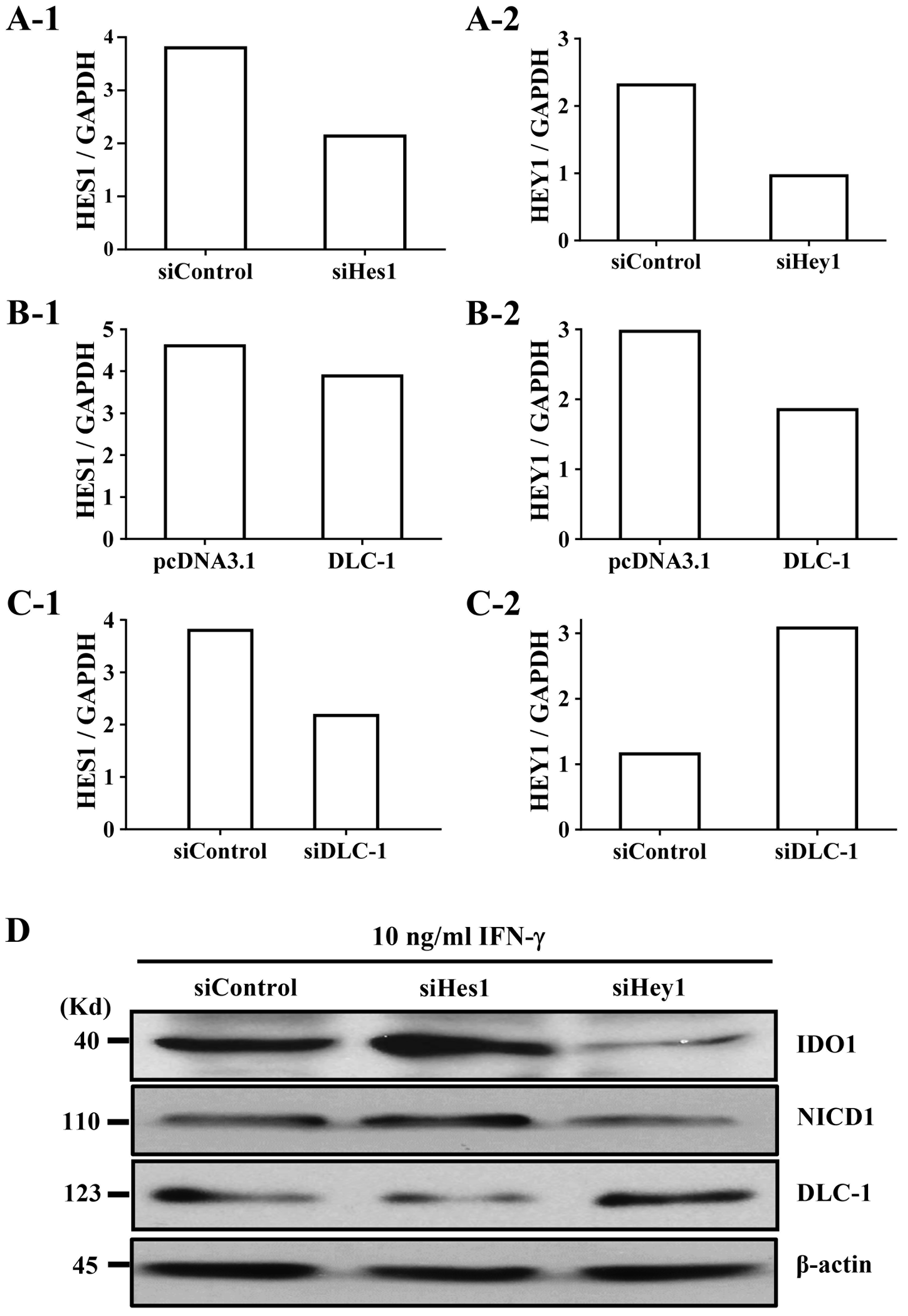

Supplement: Supplementary file 1 — Additional file 1: Figure S1. Hey1, but not Hes1, mediate the mutual exclusive relationship between DLC-1 and Notch1. A-C: RT-PCR showed that the transfection with siHes1 or siHey1 can effectively reduce the gene expression of Hes1 (A-1) or Hey (A-2), respectively; the transfection with DLC-1 cDNA caused a slight reduction of Hes1 expression (B-1), but a significant reduction of Hey1 expression (B-2). In contrast, the siDLC-1 transfection resulted in a remarkable reduction of Hes1 expression (C-1), but a significant elevation of Hey1 expression (C-2). D. Western blotting showed that, while the siHes1 transfection induced an apparent increase in the expression of both IDO1 and NICD1, but a clear decrease in DLC-1 expression, the siHey1 transfection induced a remarkable decrease in the expression of both IDO1 and NICD1, but a clear increase in DLC-1 expression. [file 12885_2020_7542_MOESM1_ESM.tif]

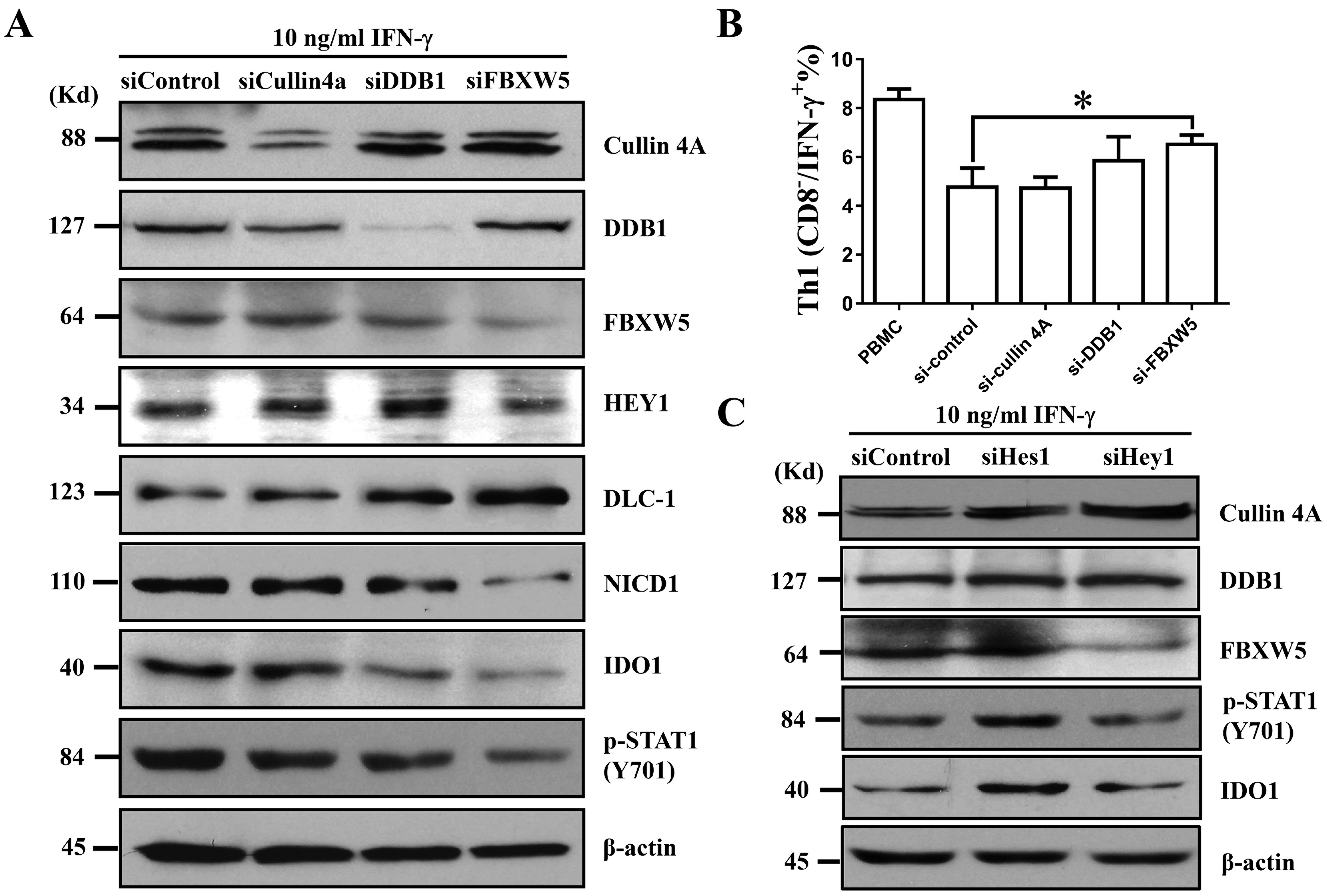

Supplement: Supplementary file 2 — Additional file 2: Figure S2. The Notch1-Hey1 axis regulates DLC-1 protein stability through modulating the expression of CULT4-DDB1-FBXW5 E3 ubiquitin ligase complex. A. Western blotting confirmed that the transfection with siCULT4, siDDB1 or siFBXW5 can effectively silence the protein expression of each target gene. The silencing of both DDB1 and FBXW5, but not CUL4A, resulted in a significant increase in DLC-1 level and a significant decrease in IFN-γ-induced IDO1 and p-STAT1 level with the silencing of FBXW5 exhibiting more significant effect than that of DDB1. The FBXW5 silencing caused in a significant decrease in both NICD1 and Hey1, while the siDDB1 silencing showed only a slight reduction of NICD1 but an increase in Hey1, and the CUL4A silencing exhibits no effect on both NICD1 and Hey1. B. The Th1 lymphocyte proliferation assay showed that the FBXW5 silencing, but not the silencing of either DDB1 or CUL4A, resulted in a significant reduction of the inhibition of Th1 lymphocyte proliferation by hUC-MSCs. C. Western blotting showed that, while the Hes1 silencing exerted no effect on the expression of CUL4A, DDB1 and FBXW5, the si-Hey1 silencing resulted in a significant reduction of FBXW5 only. [file 12885_2020_7542_MOESM2_ESM.tif]

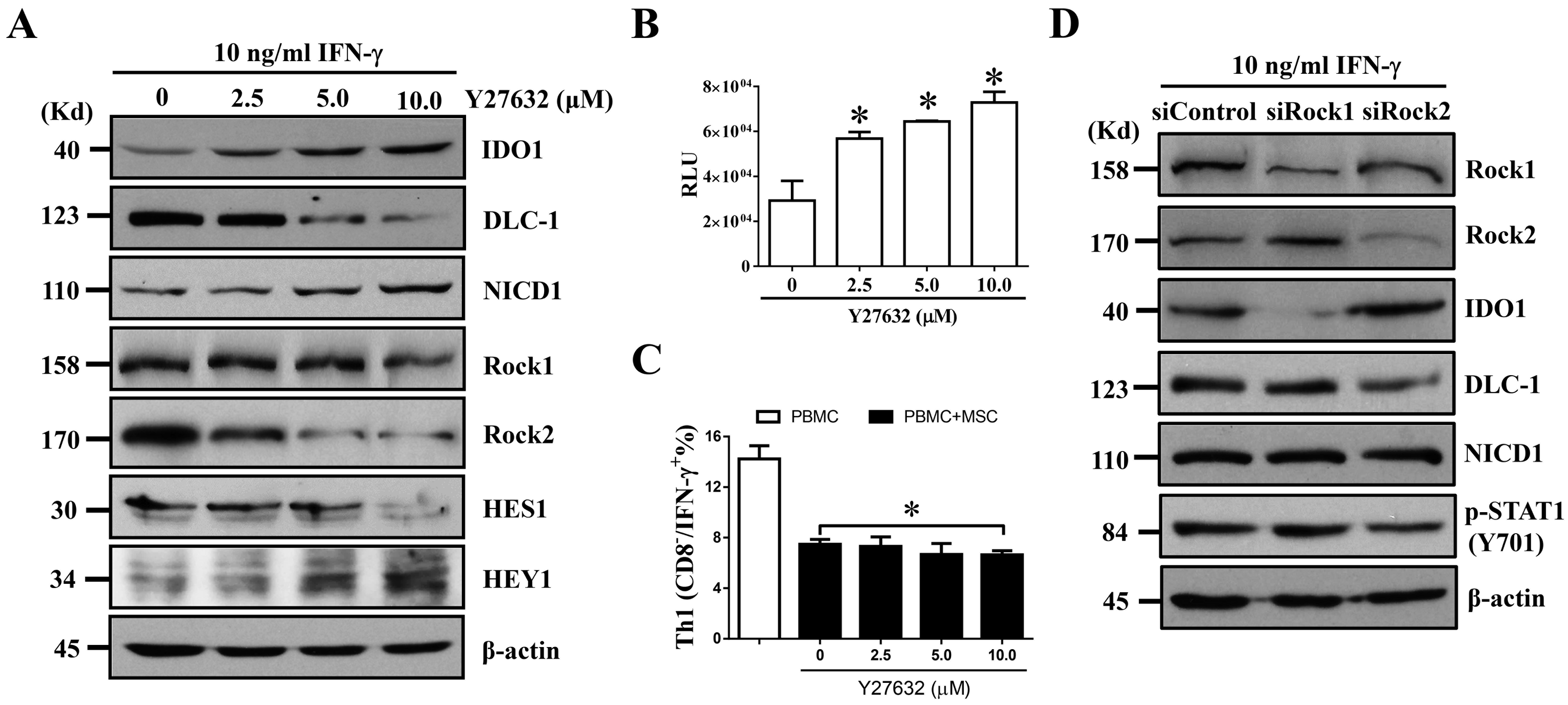

Supplement: Supplementary file 3 — Additional file 3: Figure S3. The Y27632-induced Rock2 inhibition, but not Rock1 inhibition, promotes the immunomodulation of hUC-MSCs probably through inhibiting the DLC-1 signaling while elevating the Notch1 signaling. A. Western blotting showed that the treatment with Y27632 for 24 h resulted in a dose-dependent reduction of both Rock1 and Rock2 with a much more significance seen for Rock2 as accompanied by the reduction of DLC-1 and Hes1. The treatment also resulted in a significant dose-dependent increase in the IFN-γ-induced IDO1 expression, as accompanied by the increase in both NICD1 and Hey1. B. The promoter activity assay showed that the Y27632 treatment caused a dose-dependent increase in IDO1 promoter activity. C. The Th1 lymphocyte proliferation assay showed that the pretreatment with Y27632 for 24 h exhibited a clear increase in the inhibition of Th1 lymphocyte proliferation with the statistical significance seen in the pretreatment with 10 μM Y27632. D. Western blotting showed that the silencing of Rock2, but not Rock1, exhibits the same effect as Y27632 on the expression of IDO1, DLC-1, NICD and Hey1. [file 12885_2020_7542_MOESM3_ESM.tif]
